# Usefulness of linked data for infectious disease events: a systematic review

**DOI:** 10.1017/S0950268823000316

**Published:** 2023-02-27

**Authors:** Emma Field, Melanie Strathearn, Christopher Boyd-Skinner, Amalie Dyda

**Affiliations:** 1 National Centre for Epidemiology and Population Health, Australian National University, Canberra, Australia; 2 Menzies School of Health Research, Charles Darwin University, Darwin, Australia; 3 School of Population Health, University of Queensland, Brisbane, Australia; 4 Australian Commission on Safety and Quality in Health Care, Sydney, Australia

**Keywords:** Epidemiology, infectious disease epidemiology, infectious disease, outbreaks, surveillance

## Abstract

Surveillance is a key public health function to enable early detection of infectious disease events and inform public health action. Data linkage may improve the depth of data for response to infectious disease events. This study aimed to describe the uses of linked data for infectious disease events. A systematic review was conducted using Pubmed, CINAHL and Web of Science. Studies were included if they used data linkage for an acute infectious disease event (e.g. outbreak of disease). We summarised the event, study aims and designs; data sets; linkage methods; outcomes reported; and benefits and limitations. Fifty-four studies were included. Uses of linkage for infectious disease events included assessment of severity of disease and risk factors; improved case finding and contact tracing; and vaccine uptake, safety and effectiveness. The ability to conduct larger scale population level studies was identified as a benefit, in particular for rarer exposures, risk factors or outcomes. Limitations included timeliness, data quality and inability to collect additional variables. This review demonstrated multiple uses of data linkage for infectious disease events. As infectious disease events occur without warning, there is a need to establish pre-approved protocols and the infrastructure for data-linkage to enhance information available during an event.

## Introduction

Infectious disease events cause significant impact around the globe [1, 2]. Surveillance is a key public health function to enable early detection of infectious disease events and inform public health action [3]. In many settings, for example Australia, surveillance systems are fragmented with data reported from numerous sources and shared responsibility across varying levels of government [4]. Rapid changes in technology have presented opportunities for improved timeliness, interoperability, analysis and interpretation of surveillance data. One example of this is data linkage.

Data linkage is the process of linking two or more datasets to provide more comprehensive information on individuals. For example, hospitalisation data can be linked to notifiable disease data to provide information on patient outcomes [5]. Data linkage can be performed using deterministic and probabilistic linkage methods or a combination or both [6]. Deterministic linkage is where a unique identifier is used for linkage, or a statistical linkage key is used from a combination of variables such as name, date of birth and sex [6]. Probabilistic linkage allows more flexibility to accommodate errors in data and calculate the likelihood of a match based on weightings from variables such as name, date of birth and address [6]. For both methods a linkage key is used to identify each record in place of identifiable data, ensuring that all identifiers are omitted from the final dataset to minimise risks to confidentiality [7].

There are numerous examples of the use of data linkage for infectious diseases. Data linkage has been used for infectious diseases for determining effectiveness and safety of routine immunisations [8], improving Indigenous status completeness of notification data [9] and improving case ascertainment for notifiable conditions [10, 11]. However, these examples are often for improving routine activities rather than for informing the response to an acute infectious disease event. Such events require a range of data to be collected and analysed rapidly to inform the response. These data may include, but are not limited to, notification, laboratory, hospitalisation, vaccination and mortality data. Typically, these data are collected through different systems, resulting in public health responders having to collect and analyse them separately.

Data linkage infrastructure has been established in many jurisdictions, and in some cases the addition of infectious disease data to these linked data sets [12, 13]. This provides a unique opportunity to use linked data for both surveillance of and response to infectious disease events. We hypothesise that linkage of routinely collected data may improve the depth of data for response to infectious disease events without additional primary data collection. We conducted a systematic review to describe the uses of linked data for infectious disease events.

## Methods

### Objectives

The objective of this review was to describe ways in which linked data has been used to assist in the response for acute infectious disease events (i.e., outbreaks/epidemics or pandemics). More specifically, this systematic review describes: the data sets used for data linkage; the study designs used; the methodologies used to link the data sets; the outcomes reported on; and methodological issues and limitations.

### Criteria for considering studies for this review

#### Types of intervention

A study conducted to illicit information about an infectious disease event using linkage of routinely collected data OR linkage of data collected for the purposes of the outbreak investigation with routinely collected data. We considered studies where electronic records were linked using a common unique identifier(s) and/or probabilistic or deterministic linkage.

#### Types of outcome measures: phenomena of interest

Acute infectious disease events (epidemic or pandemic) where a rapid public health response was required. The study may be conducted during or after the infectious disease event.

### Electronic searches

Pubmed, CINAHL and Web of Science were used to search for studies. The electronic database searches were conducted on 2 November 2021. The search was limited to studies published in 2000 or later and to studies published in English. The search terms were as follows: PubMed (‘data linkage’ OR ‘record linkage’ OR ‘linked records’ OR ‘linked data’ OR ‘linked database’) AND (outbreak OR epidemic OR pandemic OR communicable disease (MeSH Terms) OR ‘infectious disease’); Web of Science – TOPIC: ((‘data linkage’ OR ‘record linkage’ OR ‘linked records’ OR ‘linked data’ OR ‘linked database’) AND (outbreak OR epidemic OR pandemic OR ‘communicable disease’ OR ‘infectious disease’)) and CINAHL – (‘data linkage’ OR ‘record linkage’ OR ‘linked records’ OR ‘linked data’ OR ‘linked database’) AND (outbreak OR epidemic OR pandemic OR ‘communicable disease’ OR ‘infectious disease’).

### Screening

The titles and abstracts from the search were screened by EF and AD to determine if they should be included in the full text review. The full text of those articles which met the inclusion criteria was then reviewed by EF, AD, MS and CBS to determine if they met the criteria for final inclusion. The reference lists of included articles were reviewed to identify further studies for inclusion.

### Data extraction and synthesis

Data were extracted using a standard data extraction form by EF and MS. Data fields included on the data extraction form were: author, year, event, study objective, study design, data sources, method for data linkage, data linkage category (study used: (1) pre-established linked dataset only, (2) pre-established linked dataset plus linkage to another dataset or (3) data linked for the purpose of study only) outcomes and limitations specifically in regards to data linkage.

## Results

**Fig. 1.**
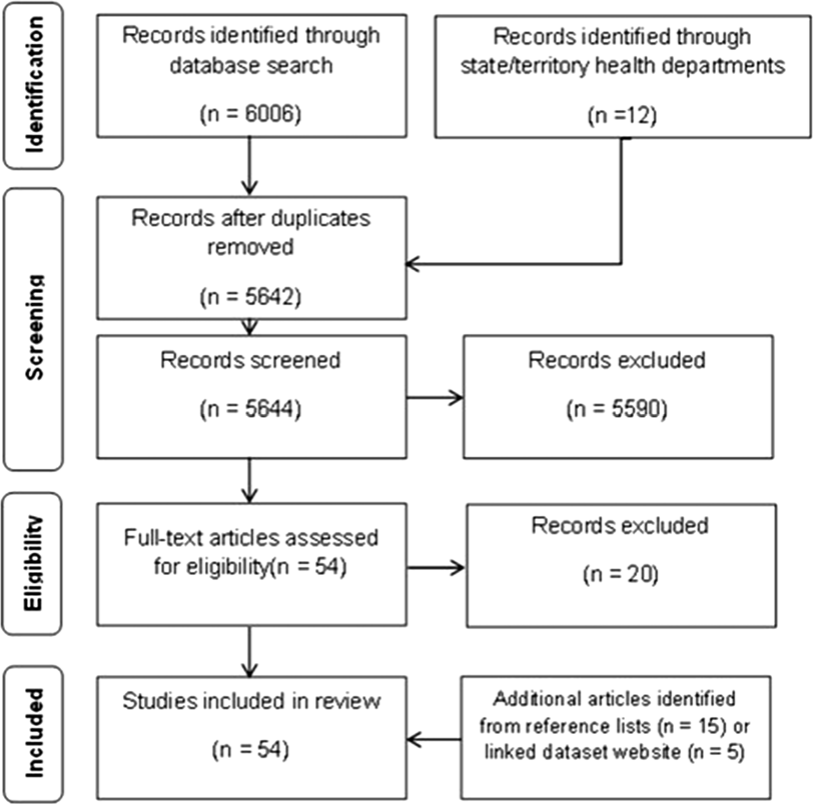
PRISMA flow diagram [91]. This figure shows the number of studies included and excluded at each stage of the review process.

A total of 6006 studies were identified from Pubmed (*n* = 5784), Web of Science (*n* = 150) and CINAHL (*n* = 72) (Fig. 1). Additionally, 12 studies were identified through contacting state and territory health departments. A total of 376 duplicates were removed. The remaining 5642 articles were screened in title and abstract review, through which 5590 were excluded. There were 54 studies for which the full text was reviewed. Twenty of these studies were excluded for the following reasons: insufficient description of the data linkage process and datasets linked [14–20]; the infectious disease event was identified as a result of the linkage rather than being initiated by the event [21]; primary data collected specifically for the event were linked rather than routinely collected data [22, 23]; a perspective paper [24], an editorial [25]; outcomes not related to an infectious disease event [26, 27]; data not linked at an individual level [28, 29]; a description of a linked dataset [30, 31]; and study protocol only [32, 33]. The editorial referred to a study which was reviewed and included [34]. Fourteen additional studies were identified through reviewing the reference lists of included articles [35–48] plus an additional five from the OPENSafely website [49–53] (Table 1).

**Table 1.** Summary of studies using data linkage for an acute infectious disease event

Author, year	Event, location	Objective category	Study design	Linkage category
MacDonald, 2006 [88]	Severe acute respiratory syndrome outbreak, Canada.	Evaluation	Review of linked records	Probabilistic
Huang, 2010 [80]	Influenza A(H1N1)pdm09 pandemic, Taiwan.	Vaccine effectiveness	Descriptive analysis	Unique identifier
Simpson, 2010 [81]	Influenza A(H1N1)pdm09 pandemic, Scotland.	Vaccine effectiveness	Cohort study	Unique identifier
Huang, 2011 [78]	Influenza A(H1N1)pdm09 pandemic, Taiwan.	Vaccine safety	Descriptive analysis	Unique identifier
Mahmud, 2011 [89]	Influenza A(H1N1)pdm09 pandemic, Canada.	Vaccine effectiveness	Case–control study	Unique identifier
Huang, 2012 [75]	Influenza A(H1N1)pdm09 pandemic, Taiwan.	Vaccine safety	Capture–recapture analysis	Unique identifier
Jules, 2012 [76]	Influenza A(H1N1)pdm09 pandemic, United States	Severity	Capture-recapture analysis	Linkage of multiple variables – not further described
Palmateer, 2012 [85]	Outbreak of anthrax infection among heroin users, Scotland.	Risk factors	Case control study	Probabilistic
Simpson, 2012 [77]	Influenza A(H1N1)pdm09 pandemic, Scotland.	Vaccine effectiveness and uptake	Cohort study with embedded case-control study	Unique identifier
Doyle, 2013 [74]	Influenza A(H1N1)pdm09 pandemic, United States.	Severity	Cohort study	Unique identifier plus other variables
Huang, 2013 [73]	Influenza A(H1N1)pdm09 pandemic, Taiwan.	Vaccine safety	Self-controlled case series	Unique identifier
Carcione, 2015 [8]	Pertussis epidemic, Western Australian, Australia.	Vaccine effectiveness	Cohort study	Probabilistic
Mahmud, 2015 [72]	Influenza A(H1N1)pdm09 pandemic, Canada.	Vaccine effectiveness	Nested case control study	Unique identifier
Sanderson, 2015 [86]	Contact investigation following diagnosis of a health care worker with infectious tuberculosis, United States.	Case finding	Contact investigation	Links of on multiple variables – not further described
Smith, 2015 [71]	Influenza A(H1N1)pdm09 pandemic, United Kingdom.	Severity	Cohort study	Links of on multiple variables – not further described
Lee, 2016 [84]	Ebola virus disease outbreak, Guinea.	Surveillance system evaluation	Sensitivity calculation	Probabilistic
Chand, 2017 [82]	*Mycobacterium chimera* associated with exposure to contaminated heater-cooler unit during cardiac surgery, United Kingdom.	Case finding	Cohort study	Unique identifier
Lee, 2018 [70]	Influenza A(H1N1)pdm09 pandemic, United Kingdom	Risk factors, severity	Cohort study	Deterministic
Robertson, 2018 [83]	*Mycobacterium chimaera* associated with exposure to contaminated heater-cooler unit during cardiac surgery, Queensland, Australia	Case finding	Case detection	Deterministic and probabilistic
Ayoubkhani, 2020 [36]	COVID-19 pandemic, England and Wales	Risk factors, severity	Cohort study	Unique identifier
Bhaskaran, 2020 [49]	COVID-19 pandemic, England	Risk factors	Cohort study	Unique identifier
Boulle 2020 [55]	COVID-19 pandemic, South Africa	Risk factors, severity	Cohort study	Unique identifier
Branden, 2020 [37]	COVID-19 pandemic, Stockholm, Sweden	Risk factors, severity	Cohort study	Unique identifier
Clift, 2020 [38]	COVID-19 pandemic, England	Risk factors, severity	Cohort study	Unique identifier
Drefahl, 2020 [47]	COVID-19 pandemic, Sweden	Risk factors	Cohort study	Unique identifier
Gobbato, 2020 [57]	COVID-19 pandemic, Northern Italy	Risk factors, severity	Cohort study	Unique identifier
Hollinghurst, 2020 [59]	COVID-19 pandemic, Wales	Severity	Cross-sectional and cohort study	Unique identifier
Liu, 2020 [60]	COVID-19 pandemic, New South Wales, Australia	Severity	Descriptive analysis	Probabilistic
Peach, 2020 [69]	COVID-19 pandemic, England	Risk factors, severity	Cohort study	Unique identifier
Reilev, 2020 [48]	COVID-19 pandemic, Denmark	Risk factors, severity	Cohort study	Unique identifier
Rentsch, 2020 [50]	COVID-19 pandemic, England	Prevention	Cohort study	Unique identifier
Schultze, 2020 [51]	COVID-19 pandemic, England	Protective factor	Cohort studies	Unique identifier
Shah, 2020 [45]	COVID-19 pandemic, Scotland	Risk factors, severity	Cohort study	Unique identifier
Williamson, 2020 [35]	COVID-19 pandemic, England	Risk factors, severity	Cohort study	Unique identifier
Wong, 2020 [52]	COVID-19 pandemic, England	Risk factors, severity	Cohort studies	Unique identifier
Bhattacharya, 2021 [54]	COVID-19 pandemic, England	Risk factors, severity	Descriptive analysis	Unique identifier
Burton, 2021 [56]	COVID-19 pandemic, Scotland	Risk factors	Descriptive analysis	Unique identifier
Curtis, 2021 [39]	COVID-19 pandemic, England	Vaccine uptake	Cohort study	Unique identifier
Forbes, 2021 [40]	COVID-19 pandemic, England	Risk factors	Cohort study	Unique identifier
Gaughan, 2021 [41]	COVID-19 pandemic, England and Wales	Risk factors	Cohort study	Unique identifier
Grint, 2021 [53]	COVID-19 pandemic, England	Severity	Cohort study	Unique identifier
Haas, 2021 [42]	COVID-19 pandemic, Israel	Vaccine effectiveness	Cohort study	Unique identifier
Hall, 2021 [58]	COVID-19 pandemic, England	Risk factors, severity	Cohort study	Unique identifier plus other variables
Liu, 2021 [61]	COVID-19 pandemic in New South Wales, Australia	Severity, risk factors	Cohort study.	Probabilistic
Mathur, 2021 [43]	COVID-19 in England	Risk factors, severity	Cohort study	Unique identifier
Nafilyan, 2021a [63]	COVID-19 in England	Risk factors	Cohort study	Deterministic and probabilistic and unique identifier
Nafilyan, 2021b [64]	COVID-19 in England	Risk factors	Cohort study	Unique identifier
Nafilyan, 2021c [62]	COVID-19 in England	Vaccine uptake	Cohort study	Deterministic and probabilistic and unique identifier
Nafilyan, 2021d [44]	COVID-19 in England	Risk factors, severity	Cohort study	Deterministic and probabilistic and unique identifier
Nunes, 2021 [65]	COVID-19 in Portugal	Vaccine effectiveness	Cohort study	Deterministic
Taji, 2021 [66]	COVID-19 pandemic, Canada	Risk factors, severity	Cohort study	Unique identifier plus other variable
Vasileiou, 2021 [46]	COVID-19 pandemic, Scotland	Vaccine effectiveness	Cohort study	Unique identifier
Walker, 2021 [67]	COVIV-19 pandemic, United Kingdom	Risk factors, severity	Point prevalence study	Deterministic
Welsh, 2021 [67]	COVID-19 pandemic, Australia	Risk factors, severity	Cohort study	Probabilistic

### Infectious disease event

The majority of the studies were based on the COVID-19 pandemic (*n* = 35, 64.8%) [35–69] and to a lesser extent the influenza A(H1N1) 2009 pandemic (*n* = 12, 22.2%) [70–81]. Two studies (3.7%) involved cases of *Mycobacterium chimaera* associated with exposure to contaminated heater-cooler units used during open cardiac surgery in the United Kingdom and Queensland, Australia [82, 83]. One study each was identified investigating an Ebola virus disease outbreak in Guinea [84], an anthrax outbreak among injecting drug users in Scotland [85], a case of tuberculosis in a health care worker in the United States [86], a pertussis outbreak in Western Australia [87] and an outbreak of severe acute respiratory syndrome (SARS) in Toronto, Canada [88].

### Study aims

The uses of linkage of routinely collected data for infectious disease events identified from these studies were in these broad categories: assessment of severity of disease and risk factors for specific populations (e.g. those with specific diseases (tuberculosis/HIV), rare diseases, pregnant women, infants, children); improve case finding/contact tracing investigations; determine uptake, safety and effectiveness of a vaccine during an outbreak/pandemic; and evaluate sensitivity and completeness of a surveillance system (e.g. for a notifiable disease or adverse events following vaccination).

The most common category of study aims was to assess the severity of outcomes and/or risk factors associated with infection and/or severe outcomes in the general population or specific population groups (*n* = 33, 61.1%), such as infants, pregnant women, children, people with rare autoimmune diseases or aged care residents [35, 55, 59, 60, 69–71, 74, 76, 85]. The second most common category of aims (*n* = 14, 25.9%) were associated with the safety, uptake and effectiveness of vaccines for either pandemic influenza A(H1N1) 2009 either in the general population, infants or in pregnant women [17, 72, 73, 75, 77, 78, 80, 81, 89] or a COVID-19 vaccination [39, 42, 46, 62, 65]. One of these studies specifically assessed the risk of a rare adverse event following vaccination, Guillian-Barre syndrome, in addition to other adverse events [80]. One study assessed the completeness of the adverse events reporting system in Taiwan [75]. Additionally, one study aimed to determine the effectiveness of preventing pertussis infection in infants through vaccinating new parents during a pertussis outbreak [8]. Two studies assessed the potential benefits of routinely prescribed pharmaceutical products on COVID-19 severity [50, 51] the first assessed the effect of hydroxychloroquine routinely prescribed for rheumatological disease on COVID-19 mortality; and the second assessed the association between routinely prescribed inhaled corticosteroids and COVID-19 related death in people with chronic obstructive pulmonary disease or asthma.

Two studies aimed to identify cases of *M. chimaera* associated with exposure to contaminated heater-cooler units used during open cardiac surgery in the United Kingdom and Queensland, Australia and one study aimed to identify contacts of a TB case [82, 83, 86]. One study aimed to evaluate the sensitivity of two passive surveillance systems for Ebola [84]. One study assessed the performance of a medical decision algorithm to mitigate spread of SARS from inter-facility patient transfers in Toronto, Canada [88].

### Study design

The cohort study design was most common (*n* = 38, 70.4%) [8, 35–53, 55, 57–59, 61–66, 68–71, 74, 77, 81, 82]. Five studies were descriptive analyses [54, 56, 60, 78, 80], three were case-control studies [72, 85, 89] and two studies used capture-recapture analysis [75, 76]. One study was a sensitivity calculation for a surveillance system [84]. Another study was a population-based self-controlled case series [73], one was a review of linked records [88], one was a retrospective case detection [83], one was a contact investigation [86] and one was a point prevalence study [67].

### Data sources

Routinely collected data sources included births, deaths, drugs misuse, notifiable diseases, hospitalisations, primary care, laboratory, pharmacy, national call centre, HIV and AIDS reporting, surveillance systems, disease registers, obstetrics, adverse drug reaction reporting, demographic databases, vaccination, patient transfer data (Table 1).

### Methods of linkage

For the majority of studies, data linkage occurred for the purpose of the study (*n* = 30). However, in the more recent studies it was common that a pre-established linked database was used (*n* = 24), of which eight were from the OpenSAFELY linked dataset [35, 39, 40, 43, 50–53].

The studies described methods to link datasets in varying levels of detail. The majority of the studies referred to using a unique identifier (*n* = 37) for the linkage [35–43, 45–59, 62, 66, 69, 73–75, 77–82]. Of these studies, three used one or more variables in addition to the unique identifier for the linkage [58, 66, 74]. Seven studies referred to using probabilistic linkage only [8, 60, 61, 68, 84, 85, 88]. Four studies cited using both deterministic and probabilistic linkage methods [44, 62, 63, 83].

### Outcomes reported

The most commonly reported outcomes focused on mortality and morbidity from influenza A(H1N1) 2009 or COVID-19. The predominate outcome reported was mortality rate (*n* = 27) from either COVID-19 (*n* = 25) [35–38, 40–45, 47–53, 55, 57, 59, 61, 63–65, 68] or H1N1 (*n* = 2) [70, 71]. Other common outcomes reported (for COVID-19 and influenza A(H1N1) 2009) included hospital admission (*n* = 13) [38, 40, 42, 43, 45, 46, 48, 57, 60, 61, 65, 68, 76], ICU admission (or severe/critical status) (*n* = 8) [40, 42, 43, 45, 48, 60, 61, 68]. Six papers reported on diagnosis of COVID-19 [40, 42, 43, 53, 58, 66], two of which separated cases into symptomatic and asymptomatic [42, 58]. Two papers reported rates of ventilation from COVID-19 [60, 68], one reported rates of emergency department presentation from COVID-19 [68], one reported on COVID-19 outbreaks in care-homes [56] and one reported on community onset *vs.* hospital onset of COVID-19 infection [54]. One paper reported complications (such as onset of pneumonia) from influenza A(H1N1) 2009 infection [70] and one reported on maternal characteristic and neonatal outcomes and maternal admission to ICU (influenza A(H1N1) 2009) [74].

Outcomes related to influenza A(H1N1) 2009 vaccine uptake (*n* = 3) [77, 80, 81], effectiveness (*n* = 4) [72, 77, 81, 89] and adverse events (*n* = 4) [73, 75, 78, 80] were also commonly reported. Two papers reported uptake of COVID-19 vaccines [39, 62] and three reported effectiveness of COVID-19 vaccines [42, 46, 65].

Additional outcomes included risk of infection in infants from pertussis between vaccinated and unvaccinated parents [8], risk of infection from Mycobacterium chimera [82] and sensitivity of calls to the national call centre and to local alerts regarding Ebola [84].

### Benefits and limitations

A commonly identified benefit of these studies was the ability to study health in population-based cohorts [37, 43, 55, 61, 63, 69, 74]. The accuracy of data was also highlighted as a benefit. In one example, a study reported the use of hospital and health records to provide accurate data which is less prone to selection and recall bias [72].

The ability to conduct more in-depth or large-scale analysis, due to increased information available through linkage from multiple sources was also identified as a strength. A paper linking hospital and primary care data allowed for more detailed analyses to investigate risk factors for complications from influenza in children. The linkage of the two data sets allowed for analysis of these risk factors managed in primary care as well as the risk of hospitalisation [70]. Large scale population analyses were common in the use of data linkage to investigate COVID-19 [43, 63]. In one example COVID-19 hospitalisation rates for all of New South Wales, Australia, were investigated using notifiable disease data and hospital record data [60].

A high proportion of the studies included in this analysis did not report limitations directly related to data linkage methods or processes. However, poor quality data – characterised by incomplete data sets, missing records or unique identifiers that were discovered during the linkage process – accounted for the most significant limitation. Mismatching of unique identifiers from probabilistic linkage methods in one study [84] saw decreased efficacy in results (sensitivity and specificity of record matching was 75%). The quality of datasets used varied greatly, with some studies reporting a substantial proportion of missing data [74, 82, 84]. Importantly these three studies were the least recent in the included studies.

Another commonly reported limitation reported was the reliance of data variables available [47, 49, 51, 55–57]. As data linkage relies on data already collected, collecting additional information is not possible. For example, a study investigating the mortality among influenza A patients admitted to hospital cited that the lack of information about comorbidities or co-existing infections was a limitation. However, the authors noted that this could be addressed with linkage to other data sources [71].

Timeliness was a clear limitation identified in the included studies. Several of the studies identified in this review were published well after the event [82, 85, 88]. For example, one of the earlier studies by MacDonald *et al*. investigating a decision support tool to assist in the mitigation of the spread of SARS was conducted using data from 2003 but published in 2006 [88].

## Discussion

This systematic review demonstrates that the linkage of routinely collected administrative datasets can be used for a variety of purposes for acute infectious disease events. Most of the studies identified in this review had been conducted in relation to the COVID-19 pandemic. We identified several key benefits of linkage of routinely collected data for infectious disease events, importantly the ability to conduct larger scale population-level studies with more detailed data. However, there are limitations to these methods for the use in responding to infectious disease events. These include timeliness, data quality and relying on data already available which does not allow for the collection of new or additional information that may be required for specific studies.

In relation to infectious disease events, data linkage can provide additional data for assessment of severity of disease and risk factors. This is particularly useful for rare diseases or events affecting specific populations such as pregnant women, infants and children. A study within the United Kingdom investigated associations between ethnicity and COVID-19 mortality, made possible by the use of linked data [36]. For outbreak response, data linkage was shown to improve case finding in a number of studies. These methods could compliment traditional case finding methods, demonstrated by Sanderson *et al*. (2015) who used hospital records and immunisation records to enhance contact tracing for infectious tuberculosis, showing improved efficiency by better targeting the response [86]. Additionally, the use of data linkage has been shown to be useful to determine uptake, safety and effectiveness of vaccines during an outbreak/pandemic [39, 46, 73]. However, these types of studies generally need to use pre-established linked data to provide findings in a timely manner [39, 46].

The primary benefit of data linkage is that population level datasets can be used allowing for population-based studies, whereby rare outcomes, exposures and risk factors can be studied. For example, the risk of Guillain-Barre syndrome after administration of the influenza A(H1N1) pandemic vaccine [80], and quantifying the risk of death from COVID-19 in people with autoimmune rheumatic disease [69]. This method also allows for more detailed and accurate analysis, as these data are not able to be collected in a study without linkage and the collection of primary data can be both time and resource intensive.

There are several limitations to data linkage studies which need to be navigated, including data availability. These types of studies can only use the data variables that are already collected, yet other variables may be required to answer certain public health research questions. Linked datasets can be complemented with primary data collection in such instances. For example, one study identified in this review investigated whether antibodies against SARS-COV-2 are associated with a decreased symptomatic and asymptomatic reinfection [58]. Questionaries on symptoms and exposures were required to complete this study, as these data were not routinely collected.

Data linkage studies are also limited by data quality. Most commonly, studies within this review reported issues due to under-reporting [54], missing data in the original data source [82] or limitations with the linkage methods used [88]. Existing unique identifiers across multiple datasets makes linkage easier. An example of this is in Taiwan where each resident is assigned a personal identification number, which allows for ease of linkage across multiple datasets such as medical records (inpatient and outpatient), vaccination data, birth registry, household registration [73, 75, 78, 80]. Within this review, data quality was less cited as a limitation over time, particularly in relation to completeness suggesting that as data quality and linkage infrastructure improves, data linkage studies will be of higher quality.

One clear limitation of the use of data linkage for infectious disease events is timeliness. Several of the studies identified in this review were published well after the infectious disease event, resulting in the findings of the study not immediately available for the public health response [72, 74]. The data needs for infectious disease events vary based on pathogen, context and clinical and public health response needs; vary over the duration of the infectious disease event; and in some circumstances cannot be anticipated [3]. However, in line with all other preparedness activities for infectious disease events, frameworks for data linkage outlining which data sources could be linked and for what purposes, as identified in this review, would be help address this.

As noted, some of these issues may improve over time with the introduction of greater data linkage infrastructure and better interoperability of clinical information systems. In the studies included within this review, data linkage predominately occurred for the purpose of the study such as the linkage of numerous data sources including general practice data, hospitalisation data and serology data to evaluate vaccination reporting for the A(H1N1) 2009 pandemic [90]. However, this appeared to change over time with recent studies, particularly those investigating COVID-19, using pre-established linked databases [30, 55, 59].

Existing linked datasets with ongoing linkage can help with timeliness as researchers can utilise the pre-existing dataset, rather than going through the process of linkage themselves. A key example of this is the OPENSafely COVID-19 dataset, open-source electronic health records data from England which can be accessed for research and analysis purposes. A number of studies within this review utilised these data in a timely manner, highlighting the utility of such resources [35, 39, 40, 43, 50, 51, 53]. The COVID-19 pandemic has demonstrated a proof of concept that data linkage can be completed in a timelier manner. COVID-19 publications were conducted rapidly in response to the pandemic. This strengthens the case for continuing to improve infrastructure and interoperability to assist with data linkage studies for possible future pandemics and ongoing infectious disease events.

Some studies that would have been eligible for inclusion in this review may not have been identified as they may have used linked data but not stated this explicitly or used terms for data linkage not included in our search terms. Further, health authorities may use data linkage for acute public health response but not published the results of such analyses. This may mean the uses of data linkage may be underreported.

This review demonstrated that data linkage has been used to answer important public health questions that can inform action during infectious disease events. A critical barrier to the use of data linkage for informing action during an infectious disease event is the time taken to gain approval for linked data, access the data and perform the linkage. This review has identified common data sets and variables used for infectious disease events, as well as proactively developed data linkage infrastructure established specifically for infectious diseases events. As infectious disease events occur without warning, it is possible to establish pre-approved protocols for data-linkage to enhance information available on case/contact finding, severity of disease; risk factors for disease; and vaccine uptake, safety and effectiveness for use during an event.

## Data Availability

The data described in this article are available on request from the authors.
